# Validity and relative validity of a novel digital approach for 24-h dietary recall in athletes

**DOI:** 10.1186/1475-2891-13-41

**Published:** 2014-04-30

**Authors:** Lindsay B Baker, Lisa E Heaton, Kimberly W Stein, Ryan P Nuccio, Asker E Jeukendrup

**Affiliations:** 1 Gatorade Sports Science Institute, 617 W. Main St., Barrington, IL 60010, USA; 2 School of Sport and Exercise Sciences, University of Birmingham, Edgbaston, Birmingham, UK

**Keywords:** Energy intake, Carbohydrate, Protein, Dietary observations, Team sports

## Abstract

**Background:**

We developed a digital dietary analysis tool for athletes (DATA) using a modified 24-h recall method and an integrated, customized nutrient database. The purpose of this study was to assess DATA’s validity and relative validity by measuring its agreement with registered dietitians’ (RDs) direct observations (OBSERVATION) and 24-h dietary recall interviews using the USDA 5-step multiple-pass method (INTERVIEW), respectively.

**Methods:**

Fifty-six athletes (14–20 y) completed DATA and INTERVIEW in randomized counter-balanced order. OBSERVATION (n = 26) consisted of RDs recording participants’ food/drink intake in a 24-h period and were completed the day prior to DATA and INTERVIEW. Agreement among methods was estimated using a repeated measures t-test and Bland-Altman analysis.

**Results:**

The paired differences (with 95% confidence intervals) between DATA and OBSERVATION were not significant for carbohydrate (10.1%, -1.2–22.7%) and protein (14.1%, -3.2–34.5%) but was significant for energy (14.4%, 1.2–29.3%). There were no differences between DATA and INTERVIEW for energy (-1.1%, -9.1–7.7%), carbohydrate (0.2%, -7.1–8.0%) or protein (-2.7%, -11.3–6.7%). Bland-Altman analysis indicated significant positive correlations between absolute values of the differences and the means for OBSERVATION vs. DATA (r = 0.40 and r = 0.47 for energy and carbohydrate, respectively) and INTERVIEW vs. DATA (r = 0.52, r = 0.29, and r = 0.61 for energy, carbohydrate, and protein, respectively). There were also wide 95% limits of agreement (LOA) for most method comparisons. The mean bias ratio (with 95% LOA) for OBSERVATION vs. DATA was 0.874 (0.551-1.385) for energy, 0.906 (0.522-1.575) for carbohydrate, and 0.895(0.395-2.031) for protein. The mean bias ratio (with 95% LOA) for INTERVIEW vs. DATA was 1.016 (0.538-1.919) for energy, 0.995 (0.563-1.757) for carbohydrate, and 1.031 (0.514-2.068) for protein.

**Conclusion:**

DATA has good relative validity for group-level comparisons in athletes. However, there are large variations in the relative validity of individuals’ dietary intake estimates from DATA, particularly in athletes with higher energy and nutrient intakes. DATA can be a useful athlete-specific, digital alternative to conventional 24-h dietary recall methods at the group level. Further development and testing is needed to improve DATA’s validity for estimations of individual dietary intakes.

## Background

An athlete’s daily nutritional intake can have a substantial impact on his/her health and performance [1,2]. Therefore, sports health professionals, such as registered dietitians (RDs), work with athletes to develop daily eating strategies [1]. To analyze an athlete’s nutrient intake, RDs typically rely upon conventional methods such as food frequency questionnaires, food logs, or 24-h dietary recall interviews [2,3]. However, conventional diet assessment techniques and nutrient databases have limitations, especially when applying them to unique populations such as athletes. For instance, conventional questionnaires and nutrient databases do not include sports nutrition products, supplements and ergogenic aids. Furthermore, due to athletes’ busy lifestyles, it is often difficult to obtain complete 3-day food records and the amount of time they have available for a consultation with a RD may be limited. In these instances it may be desirable to use a method that is tailored to the athlete, takes into account sport-specific products, and can be administered digitally for immediate feedback. Therefore, the dietary analysis tool for athletes (DATA) digital program was developed to address these issues, incorporating a customized database of sports nutrition products, and with the capacity to generate an instant report. The DATA is based on a 24-h recall model, using a modification of the validated United States Department of Agriculture (USDA) 5-step multiple-pass method [4].

The purpose of the present study was to determine DATA’s validity and relative validity for the estimation of 24-h energy, carbohydrate, protein, total fat, water, sodium, calcium, and iron intake in 14–20 year old competitive athletes. DATA’s validity was determined by comparing the agreement between dietary intake recalled from DATA and that obtained from RDs direct observations (OBSERVATION). DATA’s relative validity was determined by comparing the agreement between dietary intake recalled from DATA and that obtained from 24-h recall interviews using the USDA 5-step multiple-pass method (INTERVIEW). USDA 5-step multiple-pass was used as the reference dietary recall method for the determination of relative validity because it has been previously validated against dietary observations and the doubly labeled water technique for energy intake in children and adults [5,6] and because it is currently the conventional recall interview method of choice for many sports RDs.

## Methods

### Ethics statement

This study was approved by the Sterling Institutional Review Board (Atlanta, GA) for the protection of human study participants. Participants and their parent/guardian were informed of the experimental procedures and associated risks before providing written informed consent.

### General design

This study consisted of 3 phases: 1) tool development and pre-testing to finalize the DATA, 2) the validation of DATA by determining its level of agreement with OBSERVATION, and 3) DATA relative validity testing to determine the DATA’s agreement with INTERVIEW. The dietary intake determined from the OBSERVATION, DATA, and INTERVIEW were from the same 24-h time period (from the time the participant woke up on one day to the same time on the next day, e.g., 6:00 am to 6:00 am).

### Study participants

A total of 87 male and female competitive athletes between 14 and 20 years of age volunteered to participate in this study. Nineteen participants completed the pre-testing and 56 participants completed the validity/relative validity testing procedures. The remaining 12 participants were non-adherent with instructions for completing one or more validity/relative validity testing sessions (DATA, INTERVIEW, or OBSERVATION, or all three) and thus their data were excluded from analyses (see Quality Control for further explanation).

To be eligible to take part in this study, participants had to be participating in sports on the scholastic, collegiate, or amateur/professional/elite level. The participants competed in soccer, tennis, basketball, football, golf, lacrosse, baseball, softball, track and field, wrestling, boxing, ice hockey, figure skating, and dance (including jazz, ballet, and modern). Criteria for exclusion were extreme dietary habits, eating disorders (assessed by the SCOFF clinical prediction questions, which is a screening tool for eating disorders, [7]), current infection, and not computer literate. Participant characteristics are presented in Table 1.

**Table 1:** Participant characteristics

	**n**	**Age (yr)**	**Body mass (kg)**	**Height (cm)**
Site 1				
Male	23	16 ± 2	72.1 ± 13.6	177.5 ± 9.7
Female	9	15 ± 1	64.4 ± 7.0	169.7 ± 5.5
				
Site 2				
Male	18	16 ± 2	69.8 ± 19.0	174.4 ± 9.5
Female	6	15 ± 2	58.5 ± 8.8	163.0 ± 4.1
				
All combined	56	16 ± 2	69.4 ± 14.3	174.3 ± 9.4

Values are means ± SD. This table does not include the subjects whose data were excluded from analyses due to noncompliance (n = 12).

### Testing sites

Data collection took place at the Gatorade Sports Science Institute at IMG Academies in Bradenton, FL (Site 1) and Barrington, IL (Site 2) over a 6-week period in the months of April and May, 2012. The IMG Academies is a campus where young athletes live, train for and compete in their sport, and go to school during the academic year. The Barrington site is a traditional laboratory setting; study participants were recruited from schools in the surrounding area to come in and complete the 24-h recalls. The 24-h recalls were conducted at both sites. The dietary observations were only conducted at Site 1 because the campus setting at IMG Academies (athletes generally stayed on campus all day) enabled the researchers to observe food/drink intake for a 24-h period.

### DATA pre-testing

The purpose of the pre-testing was to obtain athletes’ feedback on DATA before finalizing the DATA. Participants were administered the DATA by a member of the study staff. After participants completed their 24-h dietary recall, they were asked to answer a questionnaire about DATA’s user-friendliness, clarity of its questions, and completeness of its food/drink options. Nineteen participants (15 male, 4 female) completed the pre-testing procedures. Some minor changes to the aesthetics, food/drink database, and portion examples of DATA were made according to the participants’ feedback. None of the 19 participants from the pre-testing procedures participated in the DATA validity testing.

### DATA relative validity testing

The relative validity of DATA was assessed by measuring its agreement with INTERVIEW. Fifty-six athletes (41 male, 15 female) across both sites completed the DATA and INTERVIEW testing. Participants were administered the DATA and INTERVIEW in a randomized counter-balanced order (back-to-back on the same day) in a private room. The consistency of the agreement between DATA and INTERVIEW was assessed with a subset of participants (selected based on participant availability/interest in further participation; n = 30; 25 male, 5 female) by repeating (RETEST) both recall methods 1–2 weeks after the first visit (TEST).

### DATA validity testing

The validity of DATA was assessed by measuring its agreement with OBSERVATION. OBSERVATION was completed with a subset of participants (selected based on participant availability/interest in participation; n = 26; 18 male, 8 female) at Site 1 only, the day prior to completing their DATA and INTERVIEW.

### Dietary observations

To determine what the participants actually ate in a 24-h period their dietary intake was observed and recorded. OBSERVATIONs were completed by 3 trained RDs. Each RD observed 1 or 2 participants at a time. The participants were aware that their dietary intake was being recorded; however, they were asked to consume their usual diet. All RDs were trained on how to observe and document the participants’ dietary intake. Observers had access to relevant information (e.g., composition and brand names) about the food/drinks served in the IMG Academies cafeterias.

On the day of the OBSERVATION, participants reported to the laboratory in the morning before eating breakfast (~6:00 am). Then, a researcher followed the participants throughout the day and observed their dietary intake from breakfast through dinner (~6:00 pm). This involved RDs sitting with the participants in the cafeteria during breakfast, lunch, and dinner, as well as attending practices/games to observe and record their entire dietary intake at these times. The RDs recorded the type and amount of foods and drinks consumed on a standardized observer’s log. To avoid being overly intrusive, portion sizes were estimated by visual observations only (no measuring spoons/cups or scales were used). Dietary intake was not observed while the participants were in their class, locker room, or dorm. Therefore, the participants were asked to take a picture of and complete a food log for any snacks or drinks consumed when they were not being observed by a RD. The participants were each given a 3 inch by 3 inch notecard with their participant identification number to include in the picture (for scale and identification purposes). For pre-packaged snacks/drinks, sports nutrition products, and supplements, participants were also asked to take a picture or turn in the nutrition facts label. The information from the RDs’ logs and the participants’ logs/pictures was entered into NutriBase (CyberSoft, Inc., Phoenix, AZ) to determine nutrient intake for the 24-h period (from the time the participants woke up on one day to the same time on the next day).

### 24-h dietary recall interview

A different investigator conducted the INTERVIEW than the one who observed the participant the previous day. During the in-person INTERVIEW, the participant was asked to describe and quantify everything consumed the previous day. The written USDA multiple-pass method with 5 passes (Quick List, Forgotten Foods List, Time and Occasion, Detail and Review, and Final Probe) was used to conduct the interview [5,6]. Food models and measuring cups/spoons were used by the investigators to illustrate examples of portion sizes. The interviewer followed a strict protocol and read questions from a script to ensure that all investigators conducted the interview in a similar manner. Answers provided by the participant were written on a standardized interview log. The information from the interviewer’s log was entered into NutriBase (CyberSoft, Inc., Phoenix, AZ) to determine the participants’ recalled nutrient intake for the 24-h period of interest (from the time the participants woke up on one day to the same time on the next day).

### Dietary analysis tool for athletes digital program

A different investigator administered DATA than the investigator that administered the INTERVIEW and the RD who observed the participant the previous day. The DATA program uses a digital platform and was administered via an application on an iPad for this study. The DATA is based on the 24-hour recall model and was designed using a modification of the USDA 5-step multiple-pass method [4]. During the administration of DATA, participants were asked to recall their dietary intake on the previous day (from the time they woke up the previous day to the same time on the day of testing, i.e., 24-h period). To enter dietary intake, athletes were asked to indicate the time, duration, and location of each meal and snack. Foods and beverages were then broken down by category (dairy, meat, fruit, vegetable, bread, etc.). Within each category, pictures and examples were used to estimate portion sizes. A separate probe was used for dietary intake during exercise. At the end of each section the investigator was cued to probe for commonly forgotten items. Upon completion of the 24-h recall, the program provides a summary of inputs, allowing for review and final revisions. The nutrient database integrated into DATA is comprised of the USDA food database augmented with full menus from large chain restaurants, sports nutrition products, and other brands and food items not available in the USDA database. Reports of 24-h nutrient intake from DATA were generated immediately upon completion of the dietary recall with the athlete and saved for subsequent comparison with INTERVIEW and OBSERVATION.

### Quality control

The RDs’ inter-observer reliability (IOR) was measured at least once per week throughout the duration of the study. To determine IOR, two RDs simultaneously observed the meals and practices of the same participant throughout the course of the day. IOR was calculated as the percent of matches [(matches/total number of items observed) X 100] between the RDs over the course of one 24-h period of observation. An item was considered a match when the RDs’ estimations agreed within one-quarter of a serving (e.g., within 2 oz for fluid and within 0.75 oz for meat). Results from IOR on 9 different participants indicated 88% mean agreement (median = 87%, minimum = 74%, maximum = 100%) across observers, which is considered acceptable [8].

As another measure of quality control of the OBSERVATION, participant adherence (following instructions to inform investigators about eating episodes outside of the RDs observation) was monitored closely to determine when an observation was incomplete. To determine whether a participant failed to inform investigators about snacking or meals not directly observed by a RD, the Principal Investigator compared the observer’s log with the corresponding interviewer’s log for all of the participants. If the participant recalled eating a snack or meal that was not on the observer’s log then that participant’s OBSERVATION data were deemed invalid/incomplete and were not included in the final data set. Nine participants failed to adhere to study instructions for the OBSERVATION testing. In many of these cases, the missing data from OBSERVATION included large snacks and/or meals consumed in the evening/before bedtime.

All of the INTERVIEWS were audio-recorded. Throughout the study, the Principal Investigator randomly selected and reviewed 10% of each interviewer’s logs and audiotapes to ensure adherence to the interview protocol. Only minor errors and deviations from the script occurred (e.g., interview pace was a little slow or minor paraphrasing of some of the interview questions), thus no INTERVIEW data were excluded from analyses. Interviewers were given immediate feedback from the Principal Investigator regarding any required changes to their interviewing style.

Regarding the participants’ adherence on DATA and INTERVIEW testing days, 3 participants only had time to complete one of the two recall methods. Another 3 participants failed to show up at all for the dietary recalls the day after OBSERVATION (these 3 participants were also 3 of the 9 that had incomplete OBSERVATION). Thus, a total of 12 participants’ data were excluded from the analyses for a non-adherence rate of 18% (12 out of the initial 68 participants enrolled for validity/relative validity testing).

### Estimated total energy expenditure

Total energy expenditure was determined by adding resting and exercise energy expenditure during the 24-h period. The athletes resting metabolic rate was estimated using the Harris-Benedict equation [9] and exercise energy expenditure was estimated using the subjects’ body mass, activity type, and activity duration, according to the tables of McArdle et al. [10]. For exercise activities not found in the McArdle et al. [10] tables, or for participants with a body mass outside of the ranges in these tables, metabolic equivalents (METs) were used as an estimate of exercise energy expenditure [11].

### Statistical methods

Centrality and typical spread of participant characteristic values (raw scores) are described by means and standard deviations (SD). For purposes of statistical assessment, all other data were log transformed to improve heterogeneity of variance. Agreement among methods (DATA vs. INTERVIEW, DATA vs. OBSERVATION, and INTERVIEW vs. OBSERVATION) was estimated using the intra-class correlation (ICC) (2-way analysis of variance, absolute agreement, average measures) and the repeated measures t-test (reported as mean percent paired difference). Ninety-five percent confidence intervals were also calculated for the ICC’s and paired differences. Mean differences of the log-transformed data in the repeated measures t-test were adjusted (100 (e^diff^ - 1)) to produce exact percent difference values.

Bland-Altman plots were prepared for energy, carbohydrate, and protein to illustrate results by individual participants and to detect any possible bias between DATA, INTERVIEW, and OBSERVATION [12]. Limits of agreement were set as 95% confidence intervals around the mean difference (line of bias), as described by Atkinson and Nevill [13]. When statistically significant correlations were observed between the absolute values of the means vs. differences (indicating non-randomness of errors) the Bland-Altman analysis was recalculated based on the natural log transformation. The natural log values of mean bias and its random error component were calculated by multiplying and dividing the antilog of mean bias by the antilog of its random error component. The mean bias ratios with 95% limits of agreement of transformed data are reported for energy, carbohydrate, and protein for each dietary assessment method comparison.

The first vs. second recall method (for the DATA vs. INTERVIEW comparison) were compared to determine whether there was an order effect of administering the recalls back-to-back. Results at Site 1 and Site 2 were compared to determine whether there were site differences in the statistical agreement between DATA and INTERVIEW. The significance level for all statistical tests was set at α = 0.05. Calculations were accomplished using Statistical Package for the Social Sciences (SPSS) version 15.0 statistical software.

## Results

Data from a total of 56 participants for INTERVIEW and DATA (32 at Site 1 and 24 at Site 2,) and 26 participants for OBSERVATION (all at Site 1) were included in the analyses. There were no order effects of the recall methods for the DATA vs. INTERVIEW comparison, as the differences (with 95% confidence intervals) between the first and second recall were not significant for energy [-34 (-230 to 163) kcal], carbohydrate [4.9 (-19.8 to 29.6) g], protein [-2.3 (-12.8 to 8.1) g], total fat [-5.0 (-17.5 to 7.5) g], water [25 (-215 to 266) mL], sodium [-406 (-820 to 7) mg], calcium [41(-66.0 to 148.3) mg], or iron [0.4 (-1.6 to 2.4) mg]. In addition, there were minimal site differences in the agreement between DATA and INTERVIEW, with the ICC for fat intake at Site 2 (0.53, -0.11 to 0.80) being the only unfavorable result. There was good agreement (significant ICCs and no paired differences) between DATA and INTERVIEW at both sites for all other nutrients.

### DATA’s validity (Agreement between DATA and OBSERVATION)

Table 2 shows the mean ± SD (untransformed data) and the statistical comparison (transformed data) between DATA and OBSERVATION for 24-h dietary intake of energy, carbohydrate, protein, total fat, water, sodium, calcium, and iron. The ICC’s were statistically significant for energy, carbohydrate, protein, total fat, water, sodium, iron, and calcium. The paired differences for carbohydrate, protein, water, sodium, and iron were not statistically significant. However, the energy, total fat, and calcium measured with DATA were significantly greater than that of OBSERVATION.

**Table 2:** Agreement between DATA and OBSERVATION (n = 26)

	**DATA mean ± SD**	**OBSERVATION mean ± SD**	**% Mean difference (95% CI)**	**p-value for % Mean difference**	**ICC (95% CI)**	**p-value for ICC**
Energy (kcal)	3498 ± 1421	3042 ± 1262	14.4 (1.2 to 29.3)*	0.033	0.83 (0.61-0.93)	0.000
Carbohydrate (g)	475 ± 190	426 ± 159	10.1 (-1.2 to 22.7)	0.080	0.85 (0.67-0.93)	0.000
Protein (g)	151 ± 59	139 ± 63	14.1 (-3.2 to 34.5)	0.111	0.82 (0.59-0.92)	0.000
Total Fat (g)	116 ± 65	91 ± 53	26.4 (6.9 to 49.6)*	0.008	0.82 (0.54-0.93)	0.000
Water (mL)	4421 ± 1222	4212 ± 1275	5.1 (-3.4 to 14.4)	0.237	0.84 (0.65-0.93)	0.000
Sodium (mg)	4672 ± 2538	4254 ± 2117	5.1 (-7.7 to 19.7)	0.435	0.91 (0.80-0.96)	0.000
Calcium (mg)	1383 ± 684	1052 ± 566	26.4 (11.1 to 44.0)*	0.001	0.90 (0.65-0.96)	0.000
Iron (mg)	22.1 ± 12.6	18.0 ± 8.0	18.4 (-0.7 to 41.3)	0.059	0.78 (0.50-0.90)	0.000

Data are from the 26 subjects who completed DATA and OBSERVATION in the TEST session. Values for DATA and OBSERVATION (shown as mean ± SD) are untransformed data. Paired comparisons and ICC analyses were conducted on transformed (natural log) data. Asterisks indicate where the mean differences are statistically significant. ICC p-values are listed as 0.000 because they are all less than 0.0005. DATA, Dietary Analysis Tool for Athletes; ICC, intraclass correlation; OBSERVATION, direct dietary observations by dietitians.

**Figure 1:**
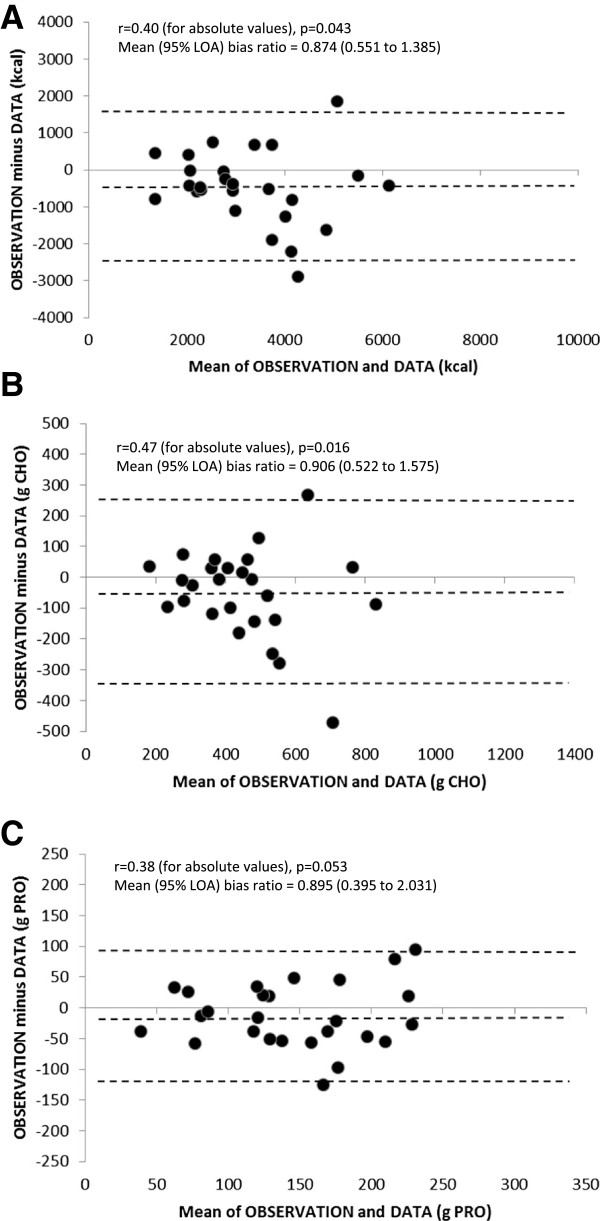
**Bland-Altman plots of OBSERVATION vs. DATA (n = 26) for energy (panel A), carbohydrate (CHO, panel B), and protein (PRO, panel C) intake.** The dashed lines represent the mean bias and 95% limits of agreement of the raw data. Correlation results between absolute values of the means vs. differences (indicating non-randomness of errors) and recalculated mean bias ratios (with 95% limits of agreement, LOA) based on transformed data are also shown for each comparison.

Figure 1 shows Bland-Altman plots of the mean of OBSERVATION and DATA against the differences between OBSERVATION and DATA for energy, carbohydrate, and protein (n = 26). The difference between OBSERVATION and DATA fell within the limits of agreement (95% CI) for all but 2 participants (1 above and 1 below the 95% CI) for energy, carbohydrate, and protein. Visual inspection of the Bland-Altman plots shows that the larger differences between OBSERVATION and DATA generally occurred at higher energy, carbohydrate, and protein ingestion rates. There were also significant correlations between the means and differences for energy (r = 0.40, p = 0.043) and carbohydrate (r = 0.47, p = 0.016), thus the Bland-Altman analysis was recalculated based on the natural log transformation. The mean bias ratios (with 95% limits of agreement) of transformed data for OBSERVATION in comparison to DATA are shown in Figure 1.

### DATA’s relative validity (Agreement between DATA and INTERVIEW)

Table 3 shows the mean ± SD (untransformed data) and the statistical comparison (transformed data) between DATA and INTERVIEW for 24-h dietary intake of energy, carbohydrate, protein, total fat, water, sodium, calcium, and iron. The ICC’s were statistically significant and the paired differences were not statistically significant for energy, carbohydrate, protein, total fat, water, sodium, iron, and calcium.

**Table 3:** Agreement between DATA and INTERVIEW (n = 56)

	**DATA mean ± SD**	**INTERVIEW mean ± SD**	**% Mean difference (95% CI)**	**p-value for % Mean difference**	**ICC (95% CI)**	**p-value for ICC**
Energy (kcal)	3315 ± 1462	3356 ± 1584	-1.1 (-9.1 to 7.7)	0.801	0.86 (0.76-0.92)	0.000
Carbohydrate (g)	449 ± 205	449 ± 216	0.2 (-7.1 to 8.0)	0.962	0.89 (0.81-0.94)	0.000
Protein (g)	140 ± 62	147 ± 77	-2.7 (-11.3 to 6.8)	0.557	0.89 (0.81-0.93)	0.000
Total Fat (g)	112 ± 73	112 ± 61	-6.1 (-17.8 to 7.4)	0.352	0.75 (0.58-0.86)	0.000
Water (mL)	4066 ± 1590	4155 ± 1672	-1.2 (-9.1 to 7.3)	0.763	0.85 (0.75-0.91)	0.000
Sodium (mg)	4594 ± 2782	4713 ± 2952	-3.2 (-13.3 to 8.1)	0.561	0.85 (0.75-0.91)	0.000
Calcium (mg)	1702 ± 1135	1628 ± 986	-0.4 (-10.0 to 10.3)	0.944	0.91 (0.85-0.95)	0.000
Iron (mg)	19.6 ± 11.1	21.1 ± 11.8	-8.4 (-18.3 to 2.7)	0.130	0.83 (0.72-0.90)	0.000

Data are from the 56 subjects who completed DATA and INTERVIEW in the TEST session. Values for DATA and INTERVIEW (shown as mean ± SD) are untransformed data. Paired comparisons and ICC analyses were conducted on transformed (natural log) data. Asterisks indicate where the mean differences are statistically significant. ICC p-values are listed as 0.000 because they are all less than 0.0005. DATA, Dietary Analysis Tool for Athletes; INTERVIEW, 24-h dietary recall interview using the USDA 5-step multiple-pass method; ICC, intraclass correlation.

**Figure 2:**
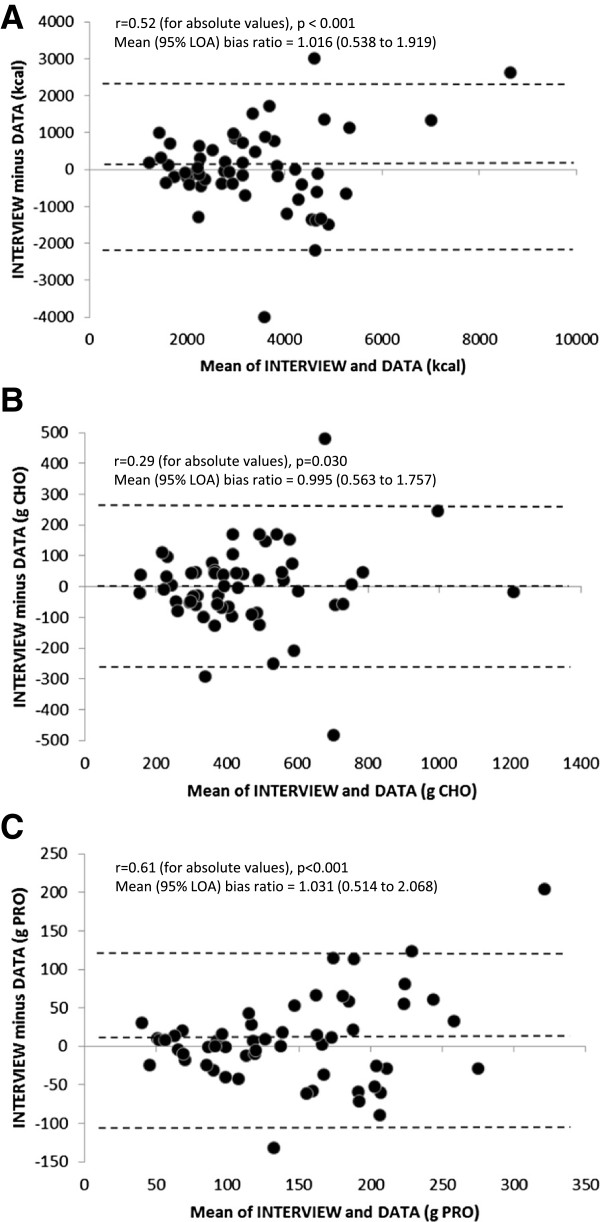
**Bland-Altman plots of INTERVIEW vs. DATA (n = 56) for energy (panel A), carbohydrate (CHO, panel B), and protein (PRO, panel C) intake.** The dashed lines represent the mean bias and 95% limits of agreement of the raw data. Correlation results between absolute values of the means vs. differences (indicating non-randomness of errors) and recalculated mean bias ratios (with 95% limits of agreement, LOA) based on transformed data are also shown for each comparison.

Figure 2 shows Bland-Altman plots of the mean of INTERVIEW and DATA against the differences between INTERVIEW and DATA for energy, carbohydrate, and protein (n = 56). The difference between INTERVIEW and DATA fell within the limits of agreement (95% CI) for all but 3 participants (2 above and 1 below the 95% CI) for energy, 2 participants (1 above and 1 below the 95% CI) for carbohydrate, and 3 participants (2 above and 1 below the 95% CI) for protein. Visual inspection of the Bland-Altman plots shows that the larger differences between INTERVIEW and DATA occurred at higher energy, carbohydrate, and protein ingestion rates. There were also significant correlations between the means and differences for energy (r = 0.52, p < 0.001), carbohydrate (r = 0.29, p = 0.030), and protein (r = 0.61, p < 0.001), thus the Bland-Altman analysis was recalculated based on the natural log transformation. The mean bias ratios (with 95% limits of agreement) of transformed data for INTERVIEW in comparison to DATA are shown in Figure 2.

Table 4 shows the ICC’s and the paired differences between DATA and INTERVIEW for the 30 participants that completed both TEST and RETEST sessions. The ICCs were statistically significant for energy, carbohydrate, protein, total fat, water, sodium, iron, and calcium for both TEST and RETEST. The paired differences between DATA and INTERVIEW were not statistically significant during TEST or RETEST for energy, carbohydrate, sodium, iron, and calcium. However, the paired differences were statistically significant for RETEST total fat and water and TEST protein in that values obtained with DATA were significantly less than that of INTERVIEW.

**Table 4:** Agreement between DATA and INTERVIEW during the TEST and RETEST sessions (n = 30)

	**TEST SESSION**					
	**DATA mean ± SD**	**INTERVIEW mean ± SD**	**% Mean difference (95% CI)**	**p-value for % Mean difference**	**ICC (95% CI)**	**p-value for ICC**
Energy (kcal)	2995 ± 1029	3278 ± 1217	-7.1 (-15.3 to 1.9)	0.116	0.86 (0.70-0.93)	0.000
Carbohydrate (g)	426 ± 152	452 ± 181	-4.2 (-14.2 to 6.9)	0.427	0.83 (0.64-0.92)	0.000
Protein (g)	126 ± 49	144 ± 69	-9.2 (-17.4 to -0.2)*	0.046	0.91 (0.81-0.96)	0.000
Total Fat (g)	93 ± 42	104 ± 40	-11.0 (-22.0 to 1.5)	0.081	0.76 (0.50-0.89)	0.000
Water (mL)	3705 ± 1674	4020 ± 1904	-7.1 (-17.8 to 5.1)	0.233	0.86 (0.70-0.93)	0.000
Sodium (mg)	4245 ± 2305	4480 ± 2474	-5.8 (-16.1 to 5.7)	0.297	0.89 (0.78-0.95)	0.000
Calcium (mg)	1804 ± 1165	1775 ± 1025	-3.3 (-17.0 to 12.7)	0.660	0.89 (0.76-0.95)	0.000
Iron (mg)	18.6 ± 9.1	22.5 ± 13.1	-14.8 (-28.3 to 1.3)	0.068	0.74 (0.46-0.88)	0.000
	**RETEST SESSION**					
	**DATA mean ± SD**	**INTERVIEW mean ± SD**	**% Mean difference (95% CI)**	**p-value for % Mean difference**	**ICC (95% CI)**	**p-value for ICC**
Energy (kcal)	2851 ± 1102	2886 ± 1128	-1.0 (-6.5 to 4.9)	0.725	0.98 (0.95-0.99)	0.000
Carbohydrate (g)	404 ± 162	394 ± 169	2.0 (-4.9 to 9.5)	0.565	0.96 (0.92-0.98)	0.000
Protein (g)	124 ± 57	127 ± 56	0.3 (-9.3 to 11.0)	0.947	0.95 (0.90-0.98)	0.000
Total Fat (g)	88 ± 50	94 ± 47	-10.7 (-19.9 to -0.4)*	0.042	0.93 (0.85-0.97)	0.000
Water (mL)	3451 ± 1388	3820 ± 1599	-9.5 (-16.6 to -1.8)*	0.018	0.93 (0.83-0.97)	0.000
Sodium (mg)	4000 ± 2002	4064 ± 1780	-4.2 (-13.9 to 6.5)	0.410	0.95 (0.89-0.97)	0.000
Calcium (mg)	1454 ± 732	1420 ± 812	6.9 (-11.7 to 29.4)	0.481	0.86 (0.71-0.93)	0.000
Iron (mg)	17.7 ± 10.7	18.3 ± 11.5	-2.6 (-17.4 to 14.8)	0.742	0.86 (0.71-0.93)	0.000

Data are from the 30 subjects who completed both TEST and RETEST sessions. Values for DATA and INTERVIEW (shown as mean ± SD) are untransformed data. Paired comparisons and ICC analyses were conducted on transformed (natural log) data. Asterisks indicate where the mean differences are statistically significant. ICC p-values are listed as 0.000 because they are all less than 0.0005. DATA, Dietary Analysis Tool for Athletes; INTERVIEW, 24-h dietary recall interview using the USDA 5-step multiple-pass method; ICC, intraclass correlation.

### Agreement between INTERVIEW and OBSERVATION

Table 5 shows the mean ± SD (untransformed data) and the statistical comparison (transformed data) between INTERVIEW and OBSERVATION for 24-h dietary intake of energy, carbohydrate, protein, total fat, water, sodium, calcium, and iron. The ICC’s were statistically significant for energy, carbohydrate, protein, total fat, water, sodium, iron, and calcium. The paired differences were not statistically significant for energy and all nutrients except total fat, in which case INTERVIEW was significantly greater than OBSERVATION.

**Table 5:** Agreement between INTERVIEW and OBSERVATION (n = 26)

	**INTERVIEW mean ± SD**	**OBSERVATION mean ± SD**	**% Mean difference (95% CI)**	**p-value for % Mean difference**	**ICC (95% CI)**	**p-value for ICC**
Energy (kcal)	3266 ± 1388	3042 ± 1262	6.4 (-2.7 to 16.2)	0.165	0.92 (0.83-0.97)	0.000
Carbohydrate (g)	436 ± 198	426 ± 159	0.8 (-8.2 to 10.6)	0.866	0.91 (0.80-0.96)	0.000
Protein (g)	143 ± 61	139 ± 63	6.4 (-5.4 to 19.5)	0.287	0.92 (0.82-0.96)	0.000
Total Fat (g)	110 ± 54	91 ± 53	26.0 (8.2 to 46.7)*	0.005	0.83 (0.54-0.93)	0.000
Water (mL)	4039 ± 1209	4212 ± 1275	-4.9 (-12.6 to 3.5)	0.232	0.86 (0.70-0.94)	0.000
Sodium (mg)	4507 ± 2504	4254 ± 2117	1.2 (-10.6 to 14.5)	0.849	0.92 (0.82-0.96)	0.000
Calcium (mg)	1192 ± 635	1052 ± 566	15.3 (-1.6 to 35.0)	0.075	0.88 (0.74-0.95)	0.000
Iron (mg)	20.3 ± 9.9	18.0 ± 8.0	12.2 (-0.5 to 26.6)	0.059	0.90 (0.77-0.96)	0.000

Data are from the 26 subjects who completed INTERVIEW and OBSERVATION in the TEST session. Values for INTERVIEW and OBSERVATION (shown as mean ± SD) are untransformed data. Paired comparisons and ICC analyses were conducted on transformed (natural log) data. Asterisks indicate where the mean differences are statistically significant. ICC p-values are listed as 0.000 because they are all less than 0.0005. INTERVIEW, 24-h dietary recall interview using USDA 5-step multiple-pass method; ICC, intraclass correlation; OBSERVATION, direct dietary observations by dietitians.

**Figure 3:**
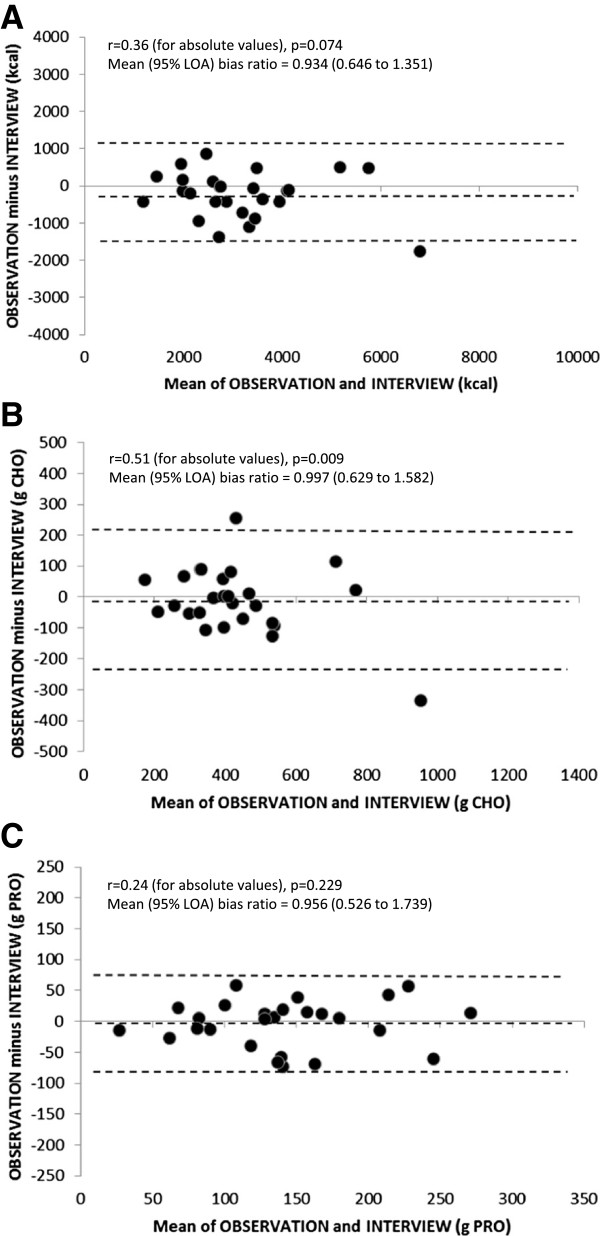
**Bland-Altman plots of OBSERVATION vs. INTERVIEW (n = 26) for energy (panel A), carbohydrate (CHO, panel B), and protein (PRO, panel C) intake.** The dashed lines represent the mean bias and 95% limits of agreement of the raw data. Correlation results between absolute values of the means vs. differences (indicating non-randomness of errors) and recalculated mean bias ratios (with 95% limits of agreement, LOA) based on transformed data are also shown for each comparison.

Figure 3 shows Bland-Altman plots of the mean of OBSERVATION and INTERVIEW against the differences between OBSERVATION and INTERVIEW for energy, carbohydrate, and protein (n = 26). The difference between OBSERVATION and INTERVIEW fell within the limits of agreement (95% CI) for all but 1 participant (below the 95% CI) for energy and 2 participants (1 above and 1 below the 95% CI) for carbohydrate. Visual inspection of the Bland-Altman plots shows that the larger differences between OBSERVATION and INTERVIEW generally occurred at higher energy, carbohydrate, and protein ingestion rates. There was also a significant correlation between the means and differences for carbohydrate (r = 0.51, p = 0.009), thus the Bland-Altman analysis was recalculated based on the natural log transformation. The mean bias ratios (with 95% limits of agreement) of transformed data for OBSERVATION in comparison to INTERVIEW are shown in Figure 3.

### Estimated total energy expenditure

The estimated energy expenditure of the 26 athletes included in the DATA vs. OBSERVATION and the INTERVIEW vs. OBSERVATION comparison was 3546 ± 959 kcal/day. The estimated energy expenditure of the 56 athletes included in the DATA vs. INTERVIEW comparison was 3135 ± 1029 kcal/day. There were no statistical differences between estimated energy expenditure and energy intake from DATA, INTERVIEW, or OBSERVATION. However, there was a tendency (p = 0.104) for the estimated energy expenditure to be greater than energy intake estimated from OBSERVATION (3042 ± 1262 kcal).

## Discussion

The main findings from this study were that in 14–20 year old competitive athletes, DATA 1) is a valid tool for determining 24-h intake of carbohydrate, protein, water, sodium, and iron at the group level, but overestimates energy, total fat, and calcium as compared to direct dietary observations, 2) has good relative validity in estimating 24-h energy, carbohydrate, protein, total fat, water, sodium, calcium, and iron intake at the group level as compared to the USDA 5-step multiple-pass dietary recall method, but 3) there are large variations in the validity and relative validity of DATA’s estimations of individual dietary intakes, particularly in athletes with higher energy and nutrient intakes.

Dietary recall is an important aspect of a RD’s consultation with athletes. However, conventional methods are not tailored specifically for athletes. A few previous studies have developed modified versions of food frequency questionnaires for athletes [14-16], but none involved a digital approach. We developed the DATA digital program with an integrated nutrient database, customized with sports nutrition products, to address these issues. The present study supports its relative validity, against the USDA 5-step multiple-pass dietary recall method, for group-level comparisons of dietary intake in 14–20 year old competitive team/skill sport athletes across two sites. Another limitation of conventional recall methods is that they do not provide immediate feedback. While the time it took to administer DATA and INTERVIEW was the same in the present study (22.3 ± 7.0 vs. 22.9 ± 6.7 min for DATA and INTERVIEW, respectively), DATA’s digital platform enabled instant output of a nutrient report, thus alleviating the time needed to complete data entry into a separate nutrient database software program as with the INTERVIEW method.

The TEST-to-RETEST consistency of DATA’s relative validity was high for energy, carbohydrate, sodium, calcium, and iron. However, the results of the present study suggested relatively low consistency in DATA’s estimations of protein, total fat, and water because the paired differences were statistically significant for RETEST total fat (-10.7%) and water (-9.5%) and TEST protein (-9.2%). Although the differences reached statistical significance it is also important to consider whether these differences were practically significant. Considering the subjective nature of dietary recall and previously reported low accuracy (up to 43% underestimation by athletes [17]) of participants’ recalled diets, it may be reasonable to conclude that ~10% difference between methods (as found with total fat, water and protein in the TEST or RETEST sessions) is acceptable. Previous validation studies have also used a 10% difference (between the novel and criterion methods) as the cutoff for determining whether a recall method was valid [5,6]. Moreover, it is commonly accepted that the participants’ ability to recall their diet may involve a learning curve and this is a limitation of all dietary recall methods. That is, participants ability to recall their diet may be relatively poor at first (during the first recall/consultation), but improve with repetition and familiarization. Similarly, it is interesting to note that the agreement between DATA and INTERVIEW generally improved (higher ICC’s and lower paired differences) from TEST to RETEST across all nutrients (see Table 4).

Compared to OBSERVATION, nutrient estimations from DATA were not statistically different for carbohydrate, protein, water, sodium, and iron and had statistically significant ICCs for energy and all nutrients measured. However, there were significant paired differences between DATA and OBSERVATION for energy, total fat, and calcium intake (see Table 2). Thus, DATA overestimates energy, total fat, and calcium estimations compared to that obtained from RD’s direct observations, suggesting future work to improve DATA in these areas may be warranted. The reason for the discrepancy between DATA and OBSERVATION is unclear. Since there were no significant mean differences between DATA and INTERVIEW, the discrepancies between DATA and OBSERVATION may not be a result of the dietary recall method or the nutrient database used in the DATA tool. Perhaps the differences between DATA and OBSERVATION could be related to the participants in the present study. The estimated 24-h energy expenditure (3546 ± 959 kcal/day) of the athletes in the DATA vs. OBSERVATION comparison (Site 1) was similar to the energy intake reported from DATA (3498 ± 1421 kcal/day). Thus, the athletes’ reported energy intake was realistic to what they may typically eat but overestimated compared to what was actually observed in the given 24-h period (3042 ± 1262). It is possible that athletes ate less than typical because they were being observed. The finding of under-eating relative to estimated energy expenditure may also be related to the fact that many of the participants were tested on competition days. Although energy expenditure tends to be higher on match days than training days [18], others have also reported that athletes tend to eat less on match days (possibly due in part to game stress) [2]. Nevertheless, more work is needed to better understand the dietary intake habits and over/under-reporting tendencies in 14–20 year old competitive athletes in stop-and-go types of activities.

When analyzing results by individual participants, the Bland-Altman plots indicate wide 95% limits of agreement for OBSERVATION vs. DATA as well as INTERVIEW vs. DATA. As shown in Figures 1 and 2, while the mean bias ratios are generally low for energy, carbohydrate, and protein, there is large variation in individual results of DATA’s validity and relative validity. In general, the greater differences between methods occurred in participants with higher energy and nutrient intakes; as confirmed by the significant correlation between the absolute values of the differences and means between OBSERVATION and DATA as well as INTERVIEW and DATA. Further development and research testing is needed to improve DATA’s validity and relative validity for estimations of energy and nutrient intake at the individual athlete level.

INTERVIEW was generally in better statistical agreement with OBSERVATION than DATA, as suggested by the slightly higher ICCs, lower paired differences, and smaller 95% limits of agreement of INTERVIEW vs. OBSERVATION compared to DATA vs. OBSERVATION (see Tables 2 and 5 and Figures 1, 2 and 3). This finding confirms that the reference method used to test the relative validity of DATA was appropriate and effective. The result of a strong agreement between INTERVIEW and OBSERVATION in the present study is consistent with previous validation studies in children and adults [4-6,19] and extends the validity of the USDA 5-step multiple-pass method when compared to observations of free-living 14–20 year old competitive athletes.

Regarding the agreement between INTERVIEW and OBSERVATION, it is also interesting to note that the athletes in the present study did not underestimate their dietary intake; as indicated by the lack of statistical paired difference between INTERVIEW and OBSERVATION for energy, carbohydrate, and protein. This finding is in contrast with previous studies in which energy intake estimations from RDs’ observations or the doubly-labeled water technique suggested participants significantly under-report their intake. For example, studies with children [20], female distance runners [21,22], gymnasts [23], ballet dancers [24], Tour de France cyclists [25], and overweight/obese adults [4] suggest that under-reporting of energy and/or macronutrient intake is common [17,26]. The discrepancy between the present and previous studies may be due to differences in the participant population (mostly stop-and-go sports in the present study vs. aesthetic or endurance sports in previous studies). Perhaps an alternative explanation for the participants’ lack of under-reporting of energy and nutrient intake is related to the methodology used in the present study. It is possible that as a result of being observed by a RD, the participants were more mindful of their dietary intake and thus made a more concentrated effort to remember what and how much they consumed. However, it was important to conduct observations in order to assess DATA’s as well as INTERVIEW’s direct validity against a gold-standard dietary assessment technique.

### Summary

Compared to direct dietary observations, the DATA digital program is a valid recall tool for estimating 24-h carbohydrate, protein, water, sodium, and iron intake at the group level. Furthermore, DATA has good relative validity for estimating 24-h energy, carbohydrate, protein, total fat, water, sodium, iron, and calcium intake at the group level when compared to the 24-h dietary interview method using the USDA’s 5-step multiple pass method. However, there are large variations in the validity and relative validity of individuals’ dietary intake estimates from DATA, particularly in athletes with higher energy and nutrient intakes. Overall, the results suggest that DATA can be a useful athlete-specific, digital alternative to conventional 24-h dietary recall methods at the group (e.g., team) level. Further development and testing is needed to improve DATA’s validity and relative validity for estimations of individual dietary intakes.

## Competing interest

Financial support for this study was provided by the Gatorade Sports Science Institute, a division of PepsiCo, Inc. Authors Lindsay B. Baker, Lisa E. Heaton, Kimberly W. Stein, Ryan P. Nuccio, and Asker E. Jeukendrup are employees of the Gatorade Sports Science Institute, a division of PepsiCo, Inc. The views expressed in this article are those of the authors and do not necessarily reflect the position or policy of PepsiCo, Inc.

## Authors’ contributions

LB designed and coordinated the study protocol and drafted the manuscript. KS conceived of the study, and participated in its design and coordination, and helped to draft the manuscript. LH is a registered dietitian and was involved in study conception, protocol design, and data collection. RN is a registered dietitian and was involved in protocol design and data collection. AE helped to interpret study results and draft the manuscript. All authors read and approved the final manuscript.
